# Complement in the Homeostatic and Ischemic Brain

**DOI:** 10.3389/fimmu.2015.00417

**Published:** 2015-08-12

**Authors:** Ali Alawieh, Andrew Elvington, Stephen Tomlinson

**Affiliations:** ^1^Neuroscience Institute, Department of Neurosciences, Medical University of South Carolina, Charleston, SC, USA; ^2^Department of Pathology and Immunology, Washington University School of Medicine, St. Louis, MO, USA; ^3^Department of Microbiology and Immunology, Ralph H. Johnson Veteran Affairs Medical Center, Medical University of South Carolina, Charleston, SC, USA

**Keywords:** complement, stroke, innate immunity, neuroprotection, brain ischemia, reperfusion injury

## Abstract

The complement system is a component of the immune system involved in both recognition and response to pathogens, and it is implicated in an increasing number of homeostatic and disease processes. It is well documented that reperfusion of ischemic tissue results in complement activation and an inflammatory response that causes post-reperfusion injury. This occurs following cerebral ischemia and reperfusion and triggers secondary damage that extends beyond the initial infarcted area, an outcome that has rationalized the use of complement inhibitors as candidate therapeutics after stroke. In the central nervous system, however, recent studies have revealed that complement also has essential roles in synaptic pruning, neurogenesis, and neuronal migration. In the context of recovery after stroke, these apparent divergent functions of complement may account for findings that the protective effect of complement inhibition in the acute phase after stroke is not always maintained in the subacute and chronic phases. The development of effective stroke therapies based on modulation of the complement system will require a detailed understanding of complement-dependent processes in both early neurodegenerative events and delayed neuro-reparatory processes. Here, we review the role of complement in normal brain physiology, the events initiating complement activation after cerebral ischemia-reperfusion injury, and the contribution of complement to both injury and recovery. We also discuss how the design of future experiments may better characterize the dual role of complement in recovery after ischemic stroke.

## Introduction

Following cerebral ischemia, most patients reperfuse at least part of the ischemic area (1), and the restoration of blood flow initiates an inflammatory cascade that causes secondary neuronal injury which can have a significant impact on functional recovery. Reperfusion can occur spontaneously, or can be achieved by surgical or pharmacological means (2). Once reperfusion occurs, there is an inflammatory reaction in which resident cells, neutrophils, macrophages, platelets, cytokines, molecular oxygen, and complement play important roles, and which culminates in necrotic and apoptotic cell death (3). The only approved pharmacological agent for the treatment of ischemic stroke is recombinant tissue-type plasminogen activator (tPA), which promotes reperfusion by enhancing the dissolution of blood clots. However, tPA must be administered with 3 h of symptom onset, and since there is a risk of uncontrollable intracranial hemorrhage, physicians are often reluctant to use this drug, with the result that only about 5% of stroke patients are treated with tPA (4, 5). There is thus a significant need for new and effective approaches to treat stroke, and reducing inflammation and secondary injury in the area surrounding the ischemic core (i.e., the penumbra) is a major therapeutic goal. Nevertheless, it is becoming increasingly clear that inflammation also has homeostatic functions within the brain, and post-ischemic inflammation is also associated with neural protection and regeneration. Therefore, understanding how inflammation is involved in the balance between neurodegenerative and neuroprotective mechanisms will be important for the development of therapeutics that optimally promote long-term functional recovery after stroke. Here, we review the role of complement in neuroinflammatory processes during brain homeostasis and in the post-ischemic brain.

In the CNS, as in other organs and tissues, complement plays a key role in the inflammatory reaction following ischemia and reperfusion. Disruption of the blood–brain barrier following ischemic stroke allows access of complement proteins to the brain, although proteins of the complement system can be locally produced by the cells of the CNS (6–8). Complement is implicated in human ischemic stroke by studies demonstrating complement activation in patients. Complement activation and deposition in areas of cerebral ischemia has also been shown in rodent models of focal cerebral ischemia, together with the upregulation of various complement proteins within the CNS. Studies utilizing complement inhibited mice or mice genetically deficient in various complement proteins have further helped elucidate the role of complement in the pathogenesis of ischemic stroke and functional recovery. However, as with other inflammatory processes, more recent studies have also documented a role for complement in homeostatic functions within the brain, and complement is also associated with post-ischemic neural protection, repair, and regeneration.

## The Complement System

**Figure 1:**
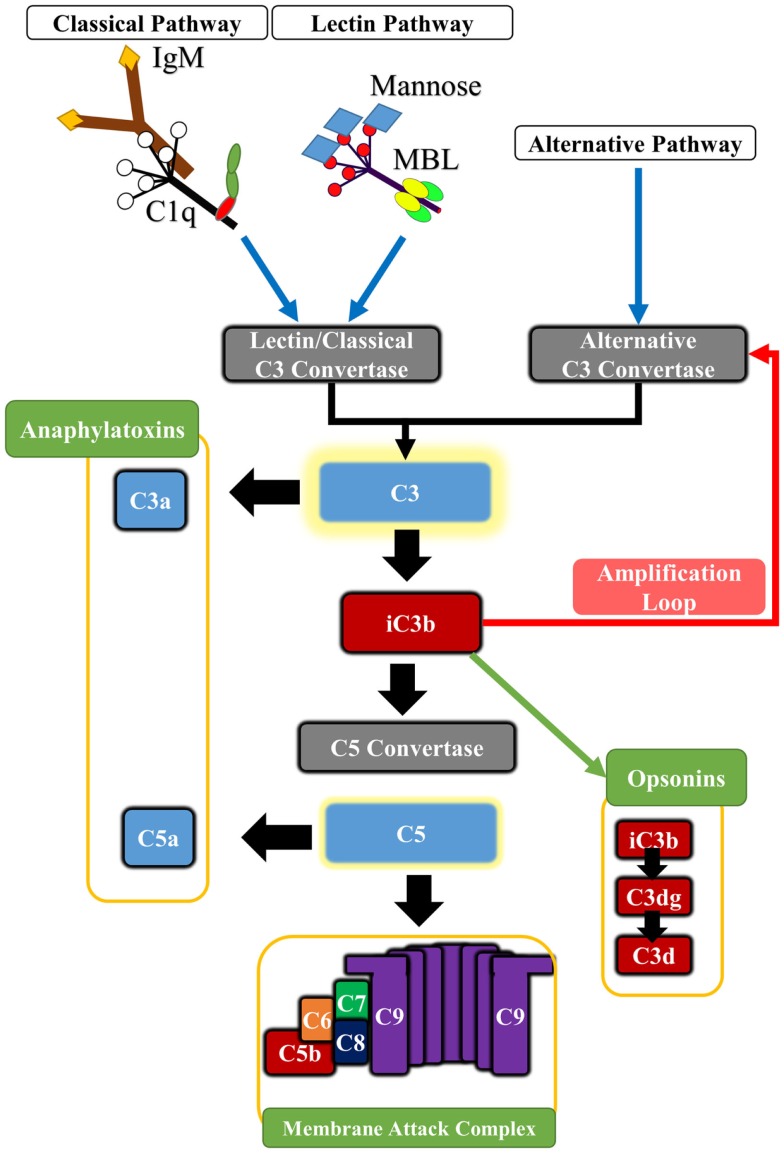
**Basic outline of the principle pathways of complement activation and complement effector molecules**. The classical pathway is triggered by the binding of C1q to antibody Fc regions, pentraxins or certain cell surface determinants. The lectin pathway is triggered by Mannose-binding lectin (MBL) or ficolins which bind to carbohydrate patterns, including those found on some IgM antibodies. Both pathways lead to cleavage of C2 and C4 components, forming the classical pathway C3 convertase (C4b2a) that cleaves C3. The alternative pathway is spontaneously activated forming the alternative pathway C3 convertase (C3bBb), and it also serves to amplify classical and lectin pathway activation. Cleavage of C3 leads to the generation of C3a and C3b. Activated C3b deposits on cell surfaces and is further degraded to iC3b, C3dg, and C3d, which serve as opsonins for receptors on immune cells. In addition, C3b associates with preformed C3 convertase, forming C5 convertase that in turn cleaves C5 into C5a and C5b. Deposition of C5b on cell surface initiates assembly of the cytolytic membrane attack complex (MAC or C5b-9). The C5a and C3a anaphylatoxins are potent pro-inflammatory molecules, and also modulate various homeostatic effects through G-protein signaling.

In addition to its well-documented role in host defense, complement plays important roles in various other physiologic and homeostatic functions, such as immune complex catabolism, the clearance of dead and dying cells, and the modulation of adaptive immune responses. Complement is also involved in various developmental and regenerative processes, and is integrated with multiple other biological systems and pathways [reviewed in Ref. (9)]. Complement activation products consisting of opsonins, anaphylatoxins, and a terminal cytolytic complex mediate the effector functions of complement. The complement cascade is activated by one of three pathways, referred to as the classical, alternative, and lectin pathways, and all pathways converge at the cleavage and activation of the complement protein C3 (Figure 1). In general, the classical pathway is activated by antibodies and apoptotic cells via recognition by C1q, and the lectin pathway is activated via recognition of carbohydrate patterns by mannose-binding lectin (MBL) or ficolins. The alternative pathway is constitutively active, and spontaneously hydrolyzed C3 can become deposited on any surface to take part in further C3 activation on that surface. An alternative pathway-activating surface is one that is unable to regulate further C3 activation and amplification. The alternative pathway also functions to amplify the classical and lectin pathways. There are, however, additional means by which complement can be activated. Extrinsic protease pathways can bypass the early pathways of complement activation and directly cleave C3 or C5 proteins; for example, thrombin can directly cleave C3 and act as a C5 convertase (10, 11). The initial products of C3 cleavage are soluble C3a and cell-bound C3b. C3b is further cleaved to yield membrane-bound iC3b, C3dg, and C3d opsonins that are recognized by receptors on immune cells. C3 cleavage also leads to C5 cleavage to yield soluble C5a and membrane-bound C5b. C5b initiates the terminal pathway and formation of the membrane attack complex (MAC) that can cause direct cell lysis, but that can also stimulate cells to release inflammatory molecules. The anaphylatoxins C3a and C5a have multiple physiologic and inflammatory activities, including the recruitment and activation leukocytes. Complement is regulated by various fluid-phase and membrane-bound inhibitors [reviewed in Ref. (12)].

## Complement in Brain Homeostasis

In contrast to systemic complement proteins that are produced predominantly by the liver, complement proteins are also be synthesized locally in the CNS. Primary studies using astrocyte and glial cell cultures demonstrated that these cells express nearly all components of the complement system (6–8). It was also shown that bacterial infection or inflammation increased expression of complement mRNA in the CNS (13, 14). Early studies focused on the protective role that the complement system played in the CNS, including sensing and response to infection, and the clearance of debris, apoptotic cells, and plaques (15–18). Further Research has also uncovered an essential role for complement in key aspects of brain function such as in synaptic pruning during CNS development, as well as other forms of synaptic plasticity (19–21). Evidence for the role of complement in elimination of synapses came from studies on the development of the retinogeniculate pathway in mice (19–21). C1q and C3 proteins produced by astrocytes were found to tag weaker synapses for removal by microglia, which drives specific innervation. This process reduces excessive connectivity between brain areas that may seed epileptogenesis as reported in C1q^−/−^ mice (22). In addition, the anaphylatoxins, C3a and C5a, have also been implicated in cerebellar development in neonatal rats. C3a and C5a receptors (C3aR and C5aR1) were transiently elevated in rat cerebellar granule neurons peaking on the 12th postnatal day (23), and agonists to these receptors transiently altered the thickness of cerebellar cortical levels. A C5a agonist caused enlargement of the external granule layer and promoted granule cell survival by inhibiting caspase-9 activity and maintaining mitochondrial integrity. A C3a agonist increased the thickness of the internal granule layer while reducing the thickness of the external granule layer, suggesting a putative role of C3a in accelerating the migration of granule cells from the external to internal granule layer (23, 24).

Components of classical and terminal complement pathways, along with the complement anaphylatoxins, have also been shown to play a role in neurogenesis and neuroprotection. The addition of C1q to rat primary neuronal cultures resulted in alteration in microRNA and mRNA expression in a direction promoting neuronal survival (25). Pathways implicated in this effect include cholesterol metabolism, synaptic function, and neuronal growth factor production. A similar neuroprotective role for C1q was reported in response to β-amyloid and serum amyloid P-induced neurotoxicity (26). Interestingly, the MAC also demonstrated a pro-survival effect on oligodendrocyte progenitor cells at sublytic concentrations. Different pathways were thought to mediate the survival and proliferative effects of sublytic MAC on oligodendrocyte progenitor cells *in vitro*, including the inhibition of caspase-3 and caspase-8 activation, upregulation of bcl-2 expression, cleavage of Bid, increase in cellular FLIP long isoform, and downregulation of FasL expression (27, 28). The use of PI3K inhibitor (LY294002) partly reversed the effects of MAC on oligodendrocyte survival, suggesting that sublytic MAC concentrations may promote downstream signaling through the PI3K pathway (28). On the other hand, it has been shown that complement receptor 2 (CR2) is expressed in adult neural progenitor cells of the dentate gyrus, and that the CR2 ligands C3d and interferon-α inhibit neural progenitor cell proliferation. Furthermore, CR2-deficient mice displayed increased basal neurogenesis, indicating that CR2 may regulate neurogenesis and that complement activation (via C3d generation) may inhibit neurogenesis (29).

**Figure 2:**
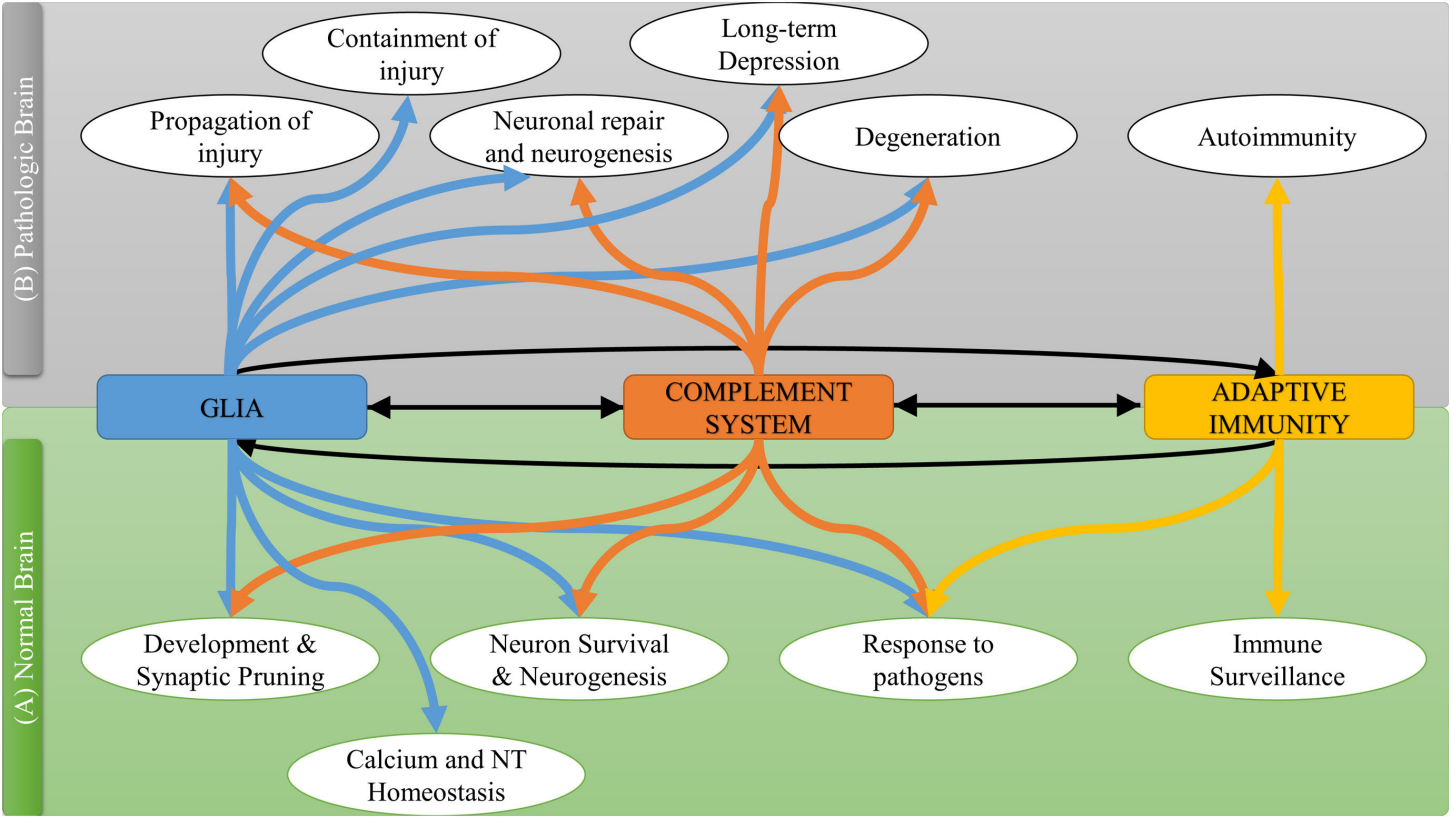
**The interplay between complement system and other immune components in normal and pathological brain**.

The C3a and C5a anaphylatoxins signal through the C3aR and C5aR1 G-protein coupled receptors, but we lack a clear understanding of their function and downstream effector mechanisms in the CNS. Certainly both anaphylatoxins can promote the production of cytokines and inflammatory mediators, and can direct and activate leukocytes, but they can also affect neuronal survival and synaptic plasticity. For example, C5aR1 is expressed on pre-synaptic terminals of mossy fibers within the hippocampus, suggesting a possible role for this receptor in synaptic/cellular plasticity (30). In addition, expression of C3aR on both neurons and glial cells suggests that C3a may play a role beyond immunological protection (31). Several studies have investigated common and different neuroprotective mechanisms of C3a and C5a. C3a was found to be involved in basal and ischemia-induced neurogenesis that was inhibited in C3-deficient mice and in mice treated with C3a receptor antagonist (C3aRA) (32). In murine primary neuronal cultures, C3a reversibly bound to neuronal cells promoting migration and differentiation through the recruitment of the intracellular ERK1/2 signaling pathway (33). C5a also recruited the ERK1/2 signaling pathway in response to glutamate-induced neurotoxicity (34). In this latter study, C5a was found to inhibit DNA fragmentation and pro-caspase-3 activation in neuronal and hippocampal cultures. Both C3a and C5a were found to increase the gene expression of neuronal growth factor in microglia, an effect mediated by C3aR and C5aR1 and inhibited by pertussis toxin (that blocks G-protein (Gi) coupled receptors) (35, 36). The increase in neuronal growth factor mRNA levels was not always associated with significant protein upregulation, but a significant synergistic effect of both anaphylatoxins with IL-1β on neuronal growth factor protein upregulation has been reported (36). Both C3a and C5a contributed to neuroprotection from glutamate-induced excitotoxicity, although each peptide had a different specific protective pattern. C3a was only protective against *N*-methyl-d-aspartate (NMDA) in the presence of astrocytes, and did not contribute to neuroprotection against Kainaite-induced excitotoxicity (37). By contrast, C5a exhibited a neuroprotective role in Kainaite rather than NMDA neurotoxicity. Also, intraventricular infusion of both Kainic acid and C5a in mice reduced caspase-3 activation and neuronal apoptosis (38), and C5aR1-deficient mice were shown to have increased susceptibility to Kainaite-induced excitotoxicity compared to wild type; treatment of wild-type mice with C5a reversed the glutamate-induced decrease in GluR2 receptor and reduced neuronal apoptosis (39). More recently, C5a was shown to reduce extracellular glutamate accumulation through the upregulation of glutamate transporter GLT-1 in microglia, suggesting a distinct mechanism of protection against excitotoxicity (40). Figure 2 summarizes the interplay between the complement system and other immune components in brain physiology and response to pathology.

## Complement in Brain Ischemia and Reperfusion Injury

The complement system has long been recognized as a potential therapeutic target for the reduction of secondary damage and improvement of outcome after stroke. Research has focused on identifying the role of complement in brain ischemia-reperfusion injury (IRI) and investigating how complement inhibition affects outcome in models of stroke. There follows a summary of studies that have been performed *in vitro*, in animal models of ischemic and hemorrhagic stroke, and in human stroke.

### Insight from *in vitro* studies

Oxygen-glucose deprivation of cultured neuronal cells is a widely used *in vitro* model for cerebral ischemia, a procedure that results in both apoptotic and necrotic cell death. Upon hypoxic insult, neuronal cultures have been shown to overexpress several complement proteins. Both mRNA and protein levels of C1q were elevated in rat neuronal cells exposed to hypoxia, and newly produced C1q preferentially deposited on hypoxic neurons, serving as both a primary opsonin and an activator of the complement cascade (41). Similarly, mouse and rat neuronal cell cultures showed increased C3 expression in response to hypoxia, a response that was shown to be associated with activation of caspase-3, a marker for apoptosis. Both C3 expression and caspase-3 activation were reduced with intravenous immunoglobulin (IVIG) treatment, suggesting that IVIG may represent an interventional therapy for stroke (42, 43). In addition, blocking C5a signaling by the use of C5aR1 antagonist or the use of neurons from C5aR1-deficient mice reduced ischemia-induced apoptosis in murine neuronal cultures indicating a pathogenic role for C5a (44, 45). The neuroprotective effect of C5aR1 antagonism could be enhanced with hypothermia without alteration in C5aR1 levels, suggesting a putative therapeutic advantage of coupling both treatments (45). On the other hand, human neurons were found to express the complement inhibitors CD59, CD46 (membrane cofactor protein) and CD55 (decay accelerating factor), and hypoxic insult neither altered inhibitor expression nor the deposition of C3d, suggesting that human neurons are protected from the effects of C3 opsonization and the MAC (46). Table 1 shows a brief summary of the different *in vitro* studies on complement involvement in experimental stroke.

**Table 1:** **Summary of *in vitro* studies on the role of complement in cerebral I/R**.

Study	Disease	Cell type	Model	Treatment	Pathway	Findings
(43)	Ischemic stroke	Mouse cortical neuron culture	Oxygen-glucose deprivation (3 h)	siRNA C3 inhibition	All pathways	(1) Inhibition of C3 expression reduced oxidative stress. (2) Ischemia promotes an increase in C3 promoter activity
(44)	Ischemic stroke	Mouse cortical neuron culture	Oxygen-glucose deprivation (12 h)	C5a C5aRA (PMX53)	All pathways	(1) C5a caused neuronal apoptosis through C5aR. (2) PMX53 blocked ischemia-induced apoptosis
(41)	Ischemic stroke	Rat PC 12 cells	Hypoxic chamber (9 h)	None	Classical pathway	(1) C1q mRNA was not expressed in PC12 but started to be expressed after hypoxia. (2) C1q protein antibody bound to the cells after hypoxia
(42)	Ischemic stroke	Rat embryo neuronal culture	Oxygen-glucose deprivation (24 h)	IVIG	All pathways	(1) C3 levels increased in cultured neurons upon OGD with a parallel increase in caspase-3. (2) IVIG reduced the levels of C3 and caspase-3 after OGD
(45)	Ischemic stroke	Astrocyte mouse culture	Oxygen-glucose deprivation (6–12 h)	C5aRA C5aR^−/−^	All pathways	(1) C5aRA after OGD and C5aR^−/−^ significantly reduced cell death and cytoplasmic LDH release. (2) Neuroprotective effect of C5aRA was enhanced upon hypothermia. (3) Expression of C5aR was not affected by hypothermia
(46)	Ischemic stroke	NT2-N human neuronal culture	1 and 0.1% hypoxia for 3 h	None	All pathways	(1) CD59 was strongly expressed on NT2-N neurons along with CD55, CD46, C3aR, and C5aR. (2) Only CD55 was affected by hypoxia with a reduction of expression. (3) Hypoxia exposure did not affect deposition of C3d

### *In vivo* studies

Animal models of ischemic stroke involve transient or permanent occlusion of the middle cerebral artery or common carotid artery, or cerebral clot embolization. Notably, the advantage of the cerebral embolization model, although more difficult and less commonly utilized, is that it better allows the evaluation of the effect of potential adjuvant therapies to tissue plasminogen activator (t-PA), the only approved treatment for acute stroke. As a plasma protease, t-PA is capable of proteolytically activating components of the complement system via the recently recognized extrinsic pathway. In support of this, an early study reported that after cerebral embolization rabbits treated with t-PA had higher levels of C3 and C5 compared to vehicle (47). Interestingly, complement depletion in the same model using cobra venom factor (CVF) did not have any effect on infarct size in the presence or absence of t-PA treatment (48). However, this study did not investigate other outcome measures that complete complement depletion may affect, and no subsequent studies have further investigated the crosstalk between t-PA and the complement system in the context of acute stroke treatment (Table 2). The use of CVF in rodent models of transient ischemia consistently demonstrates a protective effect of complement depletion. Rats subjected to bilateral transient common carotid artery occlusion and pretreated with CVF had a better outcome compared to control treated rats in terms of somatosensory evoked potentials (49). CVF also reduced infarct volume and neuronal atrophy after rat transient middle cerebral artery occlusion (MCAO), as well as after neonatal rat hypoxia (50, 51). However, in permanent ischemia rat model, CVF did not effectively reduce infract volume (52). The prominent role of reperfusion in the activation of plasma complement proteins near ischemic tissue may explain why complement depletion did not alter outcomes in models utilizing permanent ischemia.

**Table 2:** **Summary of studies investigating the role of complement in cerebral I/R using animal models**.

Study	Disease	Species	Model	Treatment	Pathway	Findings
(53)	Hemorrhagic stroke	Mouse	Intracerebral hemorrhage	C3^−/−^	All pathways	(1) C3^−/−^ had less brain edema but more cognitive and neurological deficit compared to C5^−/−^. (2) C5aR peaks at day 3 and is not expressed in sham. (3) C5aRA decreases C5aR expression and improves functional outcome alone and in combination with anti-thrombin therapy up to 5 days after surgery
				C5^−/−^		
				WT + C5aRA (PMX53)		
				WT		
(43)	Ischemic stroke	Mouse	Transient-MCAO (45 min occlusion time)	SOD1Tg^a^	All pathways	(1) SOD2KO had higher oxidative stress and increased brain C3 expression. (2) C3 expression involved microglia and neurons. (3) C3 expression was reduced by PBN compared to vehicle, and was reduced in SOD1Tg compared to WT up to 7 days after ischemia. (4) PBN reduced infarct volume and improved outcome up to 7 days after ischemia
				SOD2^−/−^		
				WT		
				WT + PBN^b^		
(44)	Ischemic stroke	Mouse	Transient- MCAO (1 h occlusion time)	CD88^−/−^ (C5aR)	All pathways	(1) C5aR^−/−^ were protected against ischemia compared to WT. (2) C5a increase post-ischemia in WT specifically in neurons. (3) C5^−/−^ had a reduced lesion volume and better neurological outcome compared to WT 24 h after reperfusion
				C5^−/−^		
				WT + C5a		
(47)	Ischemic stroke	Rabbit	Cerebral embolization	WT + t-PA	All pathways	Both C5 and C3 were significantly more activated in t-PA-treated rabbits compared to vehicle
(48)	Ischemic stroke	Rabbit	Cerebral embolization	WT + CVF^c^ +/− t-PA	All pathways	Complement depletion did not have effect on infarct size up to 7 h after embolization in any of the groups compared to controls
(54)	Ischemic stroke	Rat	Transient global cerebral ischemia (15 min)	None	Classical pathway	(1) Ischemia increased C1q mRNA expression and activity 24–72 h. (2) C1q was specifically expressed in microglia not in neurons or astrocytes
(55)	Ischemic stroke	Mouse	Transient- MCAO (45 min occlusion time)	WT + sCR1 (block C1q)	All pathways	(1) sCR1 modestly reduced acute (24 h) injury given just before ischemia. (2) sCR1-sLex markedly diminished infarct volume in a dose-dependent fashion and improved neurological outcome more than sCR1. (3) Ischemia had threefold increase in leukocyte migration that was inhibited by sCR1-Lex and not sCR1. Measurements were performed 24 h after ischemia
				WT + sCR1-sLex (block C1q and selectin)		
(56)	Ischemic stroke	Rat	Transient- MCAO (60 min occlusion time)	WT + C1 inhibitor	Classical pathway	Given reperfusion, C1 inhibitor reduced the infarct volume as well as myeloperoxidase activity 48 h after reperfusion.
(57)	Ischemic stroke	Mouse	Transient- MCAO (60 min occlusion time)	WT + CR2-Crry (30 min-postR)	All pathways	(1) C3^−/−^ and CR2-Crry-treated mice had improved survival, reduced infarct volume and expression of P-selectin and improvement of neurological outcome at 24 h after reperfusion. (2) C3^−/−^ had less apoptotic neurons than sham and CR2-Crry
				CD3^−/−^		
(58)	Ischemic stroke	Mouse	Transient- MCAO (60 min occlusion time)	None	Classical pathway	(1) C1q begins to accumulate in the brain after 6 h of ischemia. (2) C1q colocalizes with MAP2 on neurons during ischemia
(59)	Ischemic stroke	Baboons	Transient- MCAO (75 min occlusion time)	WT + sCR1	All pathways	(1) C1q blockade by sCR1 did not affect infarct volume or neurological outcome up to days after surgery. (2) C1q was deposited in the ischemic brain of vehicle-treated baboons and sCR1 was detected in the other group at 72 h after ischemia
(60)	Ischemic stroke	Mouse	Transient- MCAO (60 min occlusion time)	C1qa^−/−^	Classical and common terminal pathway	(1) C1q, C3, and C5 deposited in the ischemic side of WT but not contralaterally. (2) C1q^−/−^ did not experience improvement in cerebral infarct volumes, neurological outcome, or mortality compared to WT. (3) C3^−/−^ and C3aRA-treated WT had significant improvement in infarct volume and neurological outcome with less granulocyte infiltration and no effect on mortality. (4) C3^−/−^ improvement was reversed in C3-treated C3^−/−^ mice. (5) C5^−/−^ did not have significant change in infarct volume or mortality. Outcomes were assessed 24 h after ischemia
				C3^−/−^		
				C5^−/−^		
				WT + C3aRA		
(49)	Ischemic stroke	Rat	Bilateral common carotid artery occlusion (15 min)	WT + CVF^c^ pretreatment	Non-specific	CVF improved neurological outcome as measured by somatosensory evoked potentials up to 4 h after reperfusion
(61)	Neonatal hypoxic-ischemic encephalitis	Rat	Unilateral common carotid ligation and hypoxic atmosphere exposure	C9^−/−^ + sCR1	Classical and common terminal pathway	(1) sCR1, sCR1-sLex, or CVF did not affect the infarct volume in C9^−/−^ neonatal rats. (2) C9 administration caused a significant increase in infarct volume in C9^−/−^ compared to vehicle measured after 24 h
				C9^−/−^ + sCR1-sLex		
				C9^−/−^ + CVF^c^		
				C9^−/−^ + C9		
(50)	Neonatal hypoxic-ischemic encephalitis	Rat	Unilateral common carotid ligation and hypoxic atmosphere exposure	WT + CVF^c^	All pathways	(1) Hypoxia-induced iC3b and C9 accumulation in the brain after 16 and 24 h, respectively. (2) C3 and C9 first localized to endothelium and then to neurons. (3) CVF given 24 h before ischemia reduced infarct size without eliminating complement deposition mainly C9
(62)	Ischemic stroke	Mouse	Transient- MCAO 120 min occlusion time	WT + C1 Inhibitor	Classical pathway	(1) C1 inhibitor given after ischemia reduced focal and general deficit as well as ischemic volume at 48 h after ischemia. (2) C1 inhibitor did not stop the increase in astrocyte activation
(52)	Ischemic stroke	Rat	Permanent MCAO	WT + CVF^c^	All pathways	(1) CVF did not affect the infarct volume. (2) Infarct volume significantly increased after CRP treatment
				WT + CRP		
(63)	Ischemic stroke	Mouse	Transient- MCAO (120 min occlusion time)	WT + C1 Inhibitor	Classical pathway	(1) C1 inhibitor given at time of ischemia reduced general and focal deficits and reduced ICAM-1 and P-selectin expression. (2) C1 inhibitor also reduced pro-caspase-3 and neurofilament H expression and affected cytokine levels
(64)	Ischemic stroke	Mouse	Transient- MCAO (30 min occlusion time)	WT + C1 Inhibitor	Classical pathway	(1) C1 inhibitor reduced infarct volume in a dose-dependent fashion only when given before reperfusion. (2) C1 inhibitor also reduced general and focal deficits, neuronal apoptosis and leukocyte infiltration up to 4 days. (3) C1q^−/−^ had non-significant reduction in infarct volume, but significant reduction in ischemic volume
				C1q^−/−^		
(65)	Ischemic stroke	Mouse	Transient- MCAO (60 min occlusion time)	WT + C5aRA^d^	All pathways	C5aRA had significant reduction in infarct volume and improved neurological outcome 24 h after ischemia
(66)	Ischemic stroke	Mouse	Transient- MCAO (60 min occlusion time) and permanent MCAO	WT + C3aRA^e^	All pathways	(1) C3aRA caused smaller infarct volume less upregulation of C3aR-positive granulocytes, and less ICAM-1 protein on endothelial cells. (2) No significant change in infarct volume with C3aRA treatment 24 h after reperfusion
(67)	Hemorrhagic stroke	Mouse	Intracerebral hemorrhage	WT + C3aRA	All pathways	(1) All three groups had better performance in morris water maze compared to vehicle. (2) C5aRA only and double treatment showed significant improvement on corner turn test, better neurological score, and reduced granulocytes 72 h after surgery
				WT + C5aRA		
				WT + C3aRA and C5aRA		
(51)	Ischemic stroke	Rat	Transient MCAO (30 min) for adults and unilateral carotid artery ligation followed by hypoxia in neonates	WT + CVF^c^ pretreatment	All pathways	CVF reduced IV in adults and significantly preserved parenchymal volume in neonates and reduced cerebral atrophy 2 days after surgery
(42)	Ischemic stroke	Mouse	Transient- MCAO (60 min occlusion time) and permanent MCAO	WT + IVIG	Non-specific	(1) IVIG reduced mortality, infarct size, and functional impairment. (2) C5^−/−^ mice had improved outcome and less damage than controls, but C5aR Ant did not improve the outcome significantly. (3) IVIG significantly reduced C3b levels compared to controls as well as levels of ICAM-1, CD11a, and CD11b at 72 h post reperfusion
				WT + C5aRA		
				C5^−/−^		
(68)	Ischemic stroke	Baboons	Cerebral artery clips (75 min)	WT + sCR1-sLex	Classical pathway	(1) sCR1-Lex significantly lowered CH-50 but had no significant effect on neurological outcome. (2) sCR1-Lex-treated baboons had a larger infarct volume compared to controls
(69)	Hemorrhagic stroke	Mouse	Intracerebral hemorrhage	WT + C3aRA	Non-specific	(1) C3aRA pretreatment caused less peri-hematomal edema but no change in hematoma size and a gradual recovery of function not significant compared to vehicle. (2) C3aRA treatment post reperfusion had no significant reduction in edema or improvement of outcome up to 72 h
(70)	Ischemic stroke	Mouse	Transient- MCAO (30 min occlusion time) and permanent MCAO	WT + recombinant C1 Inhibitor (Rh C1Inh)	Classical pathway	(1) RhC1Inh but not pdC1Inh bound to MBL and was more effective inhibiting lectin pathway. (2) C1Inh reduced infarct volume if given up to 18 h post transient ischemia or within 6 h of permanent occlusion
				WT + plasma derived C1 Inhibitor (pdC1Inh)		
(71, 72)	Ischemic stroke	Mouse	Transient- MCAO (60 min occlusion time)	C3^−/−^	Classical and alternative pathway.	(1) Factor B^−/−^, C1q/MBL^−/−^, CR2-fH and CR2-Crry but not C6^−/−^ improved neurological outcome and reduced infarct volume. (2) CR2-fH was found also to reduce astrocyte reactivity and cellular apoptosis. (3) Reduction of infarct volume with CR2-fH was also seen at 7 days post reperfusion. (4) CD 59 deficiency did not affect injury after 60 min ischemia
				C6^−^/^−^		
				C1q/MBL^−/−^		
				Factor B^−/−^		
				CD59^−/−^		
				WT + CR2-fH^f^		
				WT + CR2-Crry^g^		
(73)	Ischemic stroke	Mouse	Transient- MCAO (30 min occlusion time)	WT + C3aRA	All pathways	Acute (45 min before ischemia and then daily) C3aRA treatment (1) increased neurogenesis in a dose-dependent fashion on day 7, (2) reduced T-cell infiltration, (3) reduced infarct volume, mortality and neurological deficit
(74)	Ischemic stroke	Mouse	Transient- MCAO (60 min occlusion time)	MBL^+/+^	Lectin pathway	(1) 15 min post reperfusion, CD11b increased in both neutrophils of MBL^+/+^ and MBL^−/−^ without difference but significantly more than sham. (2) There was no significant difference in infarct volume, brain edema, C3 and C1q deposition between MBL^+/+^ and MBL^−/−^ groups at 24 h after reperfusion
				MBL^−/−^		
(75)	Ischemic stroke	Mouse	Permanent MCAO	Adrenomedullin (AM)^−/−^	Alternative pathway	(1) Both AM^−/−^ and Factor H^−/−^ did not cause a significant change in infarct volume, endothelial NOS, MMP9, and COX-2 at 2 days after ischemia. (2) Bechlin-1 significantly increased after injury and together with iNOS was significantly higher in AM^−/−^ compared to WT^h^
				Factor H^−/−^		
(76)	Ischemic stroke	Mouse	Transient- MCAO (60 min occlusion time)	CD59a^−/−^	All pathways	(1) CD59a^−/−^ mice had more neurological impairment 30 min after MCAO and increased neuronal apoptosis at 1 h. (2) CD59a deficiency did not affect number of CD11b positive cells
(77)	Ischemic stroke	Mouse	Transient- MCAO (120 min occlusion time)	MBL^−/−^	Lectin pathway	(1) MBL deficiency resulted in significantly reduced infarct volume, neurological impairment and C3 deposition. (2) Reconstitution of MBL in MBL^−/−^ mice resulted in a larger infarct volume. Outcomes were measured at 48 h
				MBL^−/−^ + MBL		
(78)	Ischemic stroke	Rat	Transient- MCAO (120 min occlusion time)	WT + sCR1	Classical pathway	sCR1 significantly reduced infarct volume, myeloperoxidase activity, and C3d deposition 24 h after reperfusion
(79)	Ischemic stroke	Mouse	Neonatal hypoxia	C1q^−/−^	Classical pathway	(1) C1q^−/−^ mice had significantly lower infarct volume, improved neurological score, lower C3 deposition, and less leukocyte activation at 24 h. (2) C1q^−/−^ mice also had lower cerebral atrophy and better performance on water maze up to 8 weeks after hypoxia-ischemia
(80)	Ischemic stroke	Rat	Photochemical cortical vein occlusion	WT + C1 Inhibitor	Classical pathway	C1 Inhibitor significantly reduced infarct volume 2 h after occlusion
(32)	Ischemic stroke	Mouse	Permanent MCAO	C3^−/−^	All pathways	Both C3^−/−^ and C3aRA reduced ischemia-induced neurogenesis in the sub-ventricular zone, infarct area, and penumbra at 7 days after injury despite no significant change in infarct volume
				WT + C3aRA		
(81)	Ischemic stroke	Rat	MCAO	None	All pathways	CD11b, C1q, and C3c increased significantly in ischemic brain and remained elevated till 7 days
(82)	Ischemic stroke	Mouse	Transient- MCAO (90 min occlusion time)	MBL^−/−^	Lectin pathway	(1) MBL^−/−^ significantly reduced infarct volume and neurological deficit on days 1 and 7 after reperfusion. (2) MBL^−/−^ reduces fibrinogen accumulation in brain vasculature
				WT		
(83)	Ischemic stroke	Mouse	Transient- MCAO (60 min occlusion time)	MBL^−/−^	Lectin pathway	(1) MBL-a/c were found to accumulate in the brain after stroke peaking at 24 h. (2) MBL^−/−^ improved acute (day 1) but not subacute (day 7) measures of infarct volume and neurological deficit
				WT		
(84)	Ischemic stroke	Mouse	Permanent and transient-MCAO (30 min occlusion time)	MBL^−/−^	Lectin pathway	(1) MBL is deposited on ischemic vessels with a peak at 24 h and is associated with increased C3 deposition. (2) MBL^−/−^ mice had significantly lower infarct volume compared to WT after permanent and transient MCAO. (3) Polymannosylated dendrimers (specifically Polyman2) given up to 30 h after reperfusion significantly reduced C3 deposition, infarct volume, and neurological deficit in WT mice at 48 h only in transient MCAO
				WT		
				WT + polymannosylated dendrimers		
(84)	Ischemic stroke	Rat	Three-vessel occlusion	WT	Lectin pathway	(1) Anti-MBL-A antibody given up to 18 h after ischemia reduced neurological deficit and infarct volume at 48 h. (2) The protective effect of anti-MBL-A administration was maintained up to 28 days after ischemia
				WT + anti-MBL-A		

*Express SOD1*.

*PBN is an antioxidant*.

*CVF, cobra venom factor (deplete complements)*.

*C5aRA, C5a receptor antagonist*.

*C3aRA, C3a receptor antagonist*.

*CR2-fH, targeted inhibitor of factor H*.

*CR2-Crry, targeted inhibitor of complement activation*.

*NOS, nitric oxide synthase; COX-2, cyclo-oxygenase 2; MMP9, matrix metalloproteinase 9; iNOS, inducible nitric oxide synthase*.

Following stroke, damage to the blood–brain barrier will allow access of hematogenous complement proteins to the CNS, but there is evidence that increased local expression of complement proteins contribute to secondary injury after ischemia. For example, expression of C1q, C3, and CD11b (a component of complement receptor 3 expressed on phagocytes) is upregulated in response to ischemia in models of transient MCAO (54, 58, 60, 81). Interestingly, the increase in C1q mRNA expression was specific to microglia and did not occur in neurons or astrocytes (54), but C1q protein in the ischemic brain co-localized with the neuronal marker MAP2, suggesting that C1q is produced by microglia in response to ischemia and accumulates on neuronal cells (58). However, despite increased expression of C1q, the role of the classical pathway in post-ischemic injury is not completely clear since whereas C1q deficiency protects against murine neonatal hypoxic-ischemic brain injury (79, 85), it does not protect against murine transient MCAO (60). In neurodegenerative diseases, C1q and or C3 opsonization of synapses stimulates microglial phagocytosis, and complement-mediated synaptic clearance precedes neuronal loss (21, 86). During development, however, C1q and C3 opsonins have been shown to play an essential role in synaptic pruning and neuron remodeling (21). A feature of cerebral IRI is that it carries characteristics of both neurodegenerative disease and developmental mechanisms. Activation of inflammatory cascades after stroke causes significant secondary injury resulting in loss of both synapses and neurons in the penumbra area. Following this early insult, stroke is characterized by a window of neuroplastic response that mimics early developmental mechanisms including axonal growth and sprouting, and formation of new synapses and intra-cortical projections (87–89) [see reviews (90, 91)]. An important, yet unexplored question, is whether inhibition of the complement system after cerebral ischemia and reperfusion reduces loss of synaptic connections in the ischemic penumbra or whether this inhibition will prevent adequate synaptic pruning during rehabilitation induced cortical re-organization.

In an influential paper on the application of complement inhibition in stroke, Huang et al. described the use of soluble complement receptor 1 (sCR1) and its sialyl Lewis × glycosylated form (sCR1-sLex) to study the effect of systemic complement inhibition and selectin-targeted complement inhibition on outcomes after murine stroke. Soluble CR1 inhibits all complement pathways at the C3 activation step, and the sLex carbohydrate moiety binds to both P and E selectin, adhesion molecules that are upregulated on activated endothelium. Following MCAO and reperfusion, sCR1 modestly reduced ischemic injury while sCR1-sLex markedly diminished infarct volume, improved neurological outcome, and inhibited leukocyte migration (55). Thus, decoration sCR1 with the sLex moiety increased the efficacy of complement inhibition by targeting the inhibitor to selectin-expressing activated brain endothelial surfaces. A more recent study demonstrated that a truncated form of sCR1 also significantly improved outcome in a rat model of transient MCAO (78). Nevertheless, studies in a non-human primate stroke model failed to reproduce the earlier findings of sCR1 and sCR1-sLex treatment that was reported in mice (59). Similarly, the therapeutic value of sCR1-sLex failed to translate to baboons in a study by Ducruet et al. (66). In this latter study, sCR1-sLex reduced complement hemolytic activity, but failed to improve neurological outcome; in fact, treatment increased infarct volume in baboons. One possible explanation for this outcome is that sCR1-sLex has multivalent selectin-targeting sites and may promote homotypic platelet–platelet aggregation, and potentially thrombosis.

Another strategy used to target complement inhibition to an anatomical site is to link a complement inhibitor to a recombinant portion of complement receptor type 2 (CR2). Ligands for CR2 are C3 activation products that become covalently attached at sites of complement activation (57). Complement receptor 1-related gene/protein-y (Crry) is a rodent structural and functional analog of CR1, and a CR2-Crry construct administered 90 min after ischemia in a murine MCAO model was shown to improve survival, reduce infarct volume, reduce P-selectin expression and neutrophil recruitment, and improve neurological outcome. Acute outcomes after a single injection of CR2-Crry were comparable to those in C3-deficient mice (57). It has also been shown that targeted complement inhibition with CR2-Crry does not impair host susceptibility to infection, unlike systemic inhibition of C3 deficiency, a potentially important consideration for stroke patients who are at increased risk of infection (57).

The sCR1 and Crry inhibitors described above are pan complement inhibitors. Interventional studies in experimental models of stroke have also been performed with C1-inhibitor (C1-inh) that inhibits only the classical and lectin pathways. In various ischemic stroke models, C1-inh has been shown to decrease infarct volume (56, 62, 64, 70, 80), reduce focal and general deficits (62–64), inhibit leukocyte infiltration and myeloperoxidase activity (56, 63, 64), and diminish neuronal apoptosis (63, 64) (summarized in Table 2). Taken together with the data reported above that C1q deficiency is not protective in murine models of ischemic stroke, these data suggest a role for the lectin pathway in promoting cerebral IRI. Of note, however, all of the studies with C1-inh evaluated acute outcome after stroke, and it is possible that C1q-mediated uptake of apoptotic cells, an important anti-inflammatory and reparatory mechanism, may play a role in outcome in the subacute phase after stroke.

Further investigation has indeed demonstrated an important role for the lectin pathway in murine models of stroke. MBL deficiency resulted in significantly reduced infarct volumes, neurological impairment, and C3 deposition, whereas reconstitution of MBL-deficient mice with MBL resulted in larger infarct volumes (77). Two additional studies have also since demonstrated that MBL deficiency is protective against ischemic stroke (82, 84), and it has also been shown that an inhibitor of the lectin pathway, Polyman2, is protective when given up to 24 h after ischemia in mice (84). The same study demonstrated that an anti-MBL antibody is also protective in a rat model of stroke when given up to 18 h after ischemia, and as assessed 28 days after stroke. Thus, lectin pathway inhibition appears to be a therapeutic target with a wide therapeutic window. It should be noted, nevertheless, that Morrison et al. [(74) p. 52] found no significant difference in infarct volume, brain edema, or complement deposition between wild-type and MBL-deficient mice, and that Ducruet et al. (83) found that acute-phase protection in MBL-deficient mice was not sustained in the subacute phase. With regard to the different subacute outcomes in lectin pathway deficient vs. lectin pathway inhibited mice, an explanation could be that while acute complement activation is injurious, it also plays a role in repair and regenerative mechanisms in the subacute phase. Unlike deficiency, acute temporary lectin pathway inhibition would have minimal impact on the lectin pathway in the subacute phase.

The alternative complement pathway can be spontaneously activated, but it also serves as an amplification loop for the classical or lectin pathways. Whichever way complement is activated, it has been estimated that 80% of complement activation products can be the result of alternative pathway amplification (92). This is likely the reason that although the classical and lectin pathways can variously play essential roles in autoimmune, inflammatory, and ischemic disease models, the alternative pathway is nearly always required for full *in vivo* expression of injury (93, 94). And cerebral IRI is no exception, since although the studies reported above indicate a key role for the lectin pathway in ischemic stroke, mice deficient in factor B (alternative pathway protein) or treated with CR2-fH (targeted alternative pathway inhibitor) were protected to a similar extent as C1q/MBL-deficient mice in terms of several injury and neurological outcome measures (72). Together these data indicate that the alternative pathway is not alone sufficient to initiate complement activation, but that it acts to propagate cerebral injury via amplification of the cascade. Of note, a single CR2-fH treatment administered 90 min post ischemia resulted in significantly reduced astrocyte reactivity, cellular apoptosis and infarct volume for up to 7 days after reperfusion (72). In unpublished work, we have shown that unlike CR2-fH, CR2-Crry does not provide sustained protection into the subacute phase after stroke. This may reflect an advantage of not blocking all complement pathways in order to maintain some level of complement activation that may contribute to homeostatic or repair processes.

With regard to the terminal complement pathway, data indicate that the MAC is an important mediator of IRI in several organs and tissues, including spinal cord injury (95) and traumatic brain injury (96). In the case of cerebral IRI, however, the role of the MAC is less clear. Mice deficient in C6, a component of the MAC, are not protected from cerebral injury after 60 min MCAO and 24 h reperfusion, indicating that the MAC does not play a role in cerebral IRI (72). In agreement with this conclusion, mice deficient in CD59, a membrane-bound inhibitor of the MAC, do not display exacerbated cerebral injury in the same model (72). However, CD59-deficient mice show worse outcomes in the MCAO model with shorter ischemic times (30 min as opposed to 60 min), and thus the terminal pathway may contribute to cerebral IRI under conditions of less severe injury or ischemia (76).

Studies on the anaphylatoxins, C3a and C5a, have indicated important roles for these peptides in cerebral IRI. In a transient MCAO model, C3-deficient mice and wild-type mice treated with a C3a receptor antagonist (C3aRA) showed significant improvements in infarct volume and neurological outcomes, with less granulocyte infiltration. Furthermore, the effect was reversed when C3-deficient mice were reconstituted with C3 (60). A similar effect of C3aRA was shown in a transient MCAO model (66), but not in a model with permanent occlusion (73). The different effects of C3aRA in the two models may be related to inhibition of C3a-dependent activation of blood-derived immune cells following reperfusion. C5aR1-deficient mice are also protected against cerebral IRI following transient MCAO (44), but pretreatment of wild-type mice with C5aRA did not consistently improve neurological outcome or infarct volume (42, 65). Interestingly, C5aRA was also found to improve outcome in animal models of hemorrhagic stroke, either alone or in combination with C3aRA (53, 67, 69). These studies on the role of C3a and C5a in stroke pathogenesis were performed by analyzing acute outcomes, and it remains unclear what effect interfering with C3a- and C5a-mediated signaling would have on subacute and chronic outcomes. A concern with inhibiting C3a and C5a signaling, especially at later time points after stroke, is the effect on neurogenesis and cognitive performance, since both anaphylatoxins have implicated roles in basal and stress-induced neurogenesis (32–34). In this regard, a study using C3aRA treatment after MCAO indicated that the effect of C3a inhibition on neurogenesis is dose dependent. High-dose treatment with C3aRA (twice daily for 10 days) reduced ischemia-induced neurogenesis in the sub-ventricular zone, infarct area, and penumbra at 7 days after permanent MCAO (32). However, low dose C3aRA treatment increased neurogenesis in a dose-dependent fashion on day 7 in a transient MCAO model with 30 min ischemia (73). This may indicate that a titrated reduction of C3a-dependent signaling may limit the pro-inflammatory effects of C3a (especially in models that include reperfusion), while at the same time maintain the homeostatic effects of C3a on neurogenesis.

**Figure 3:**
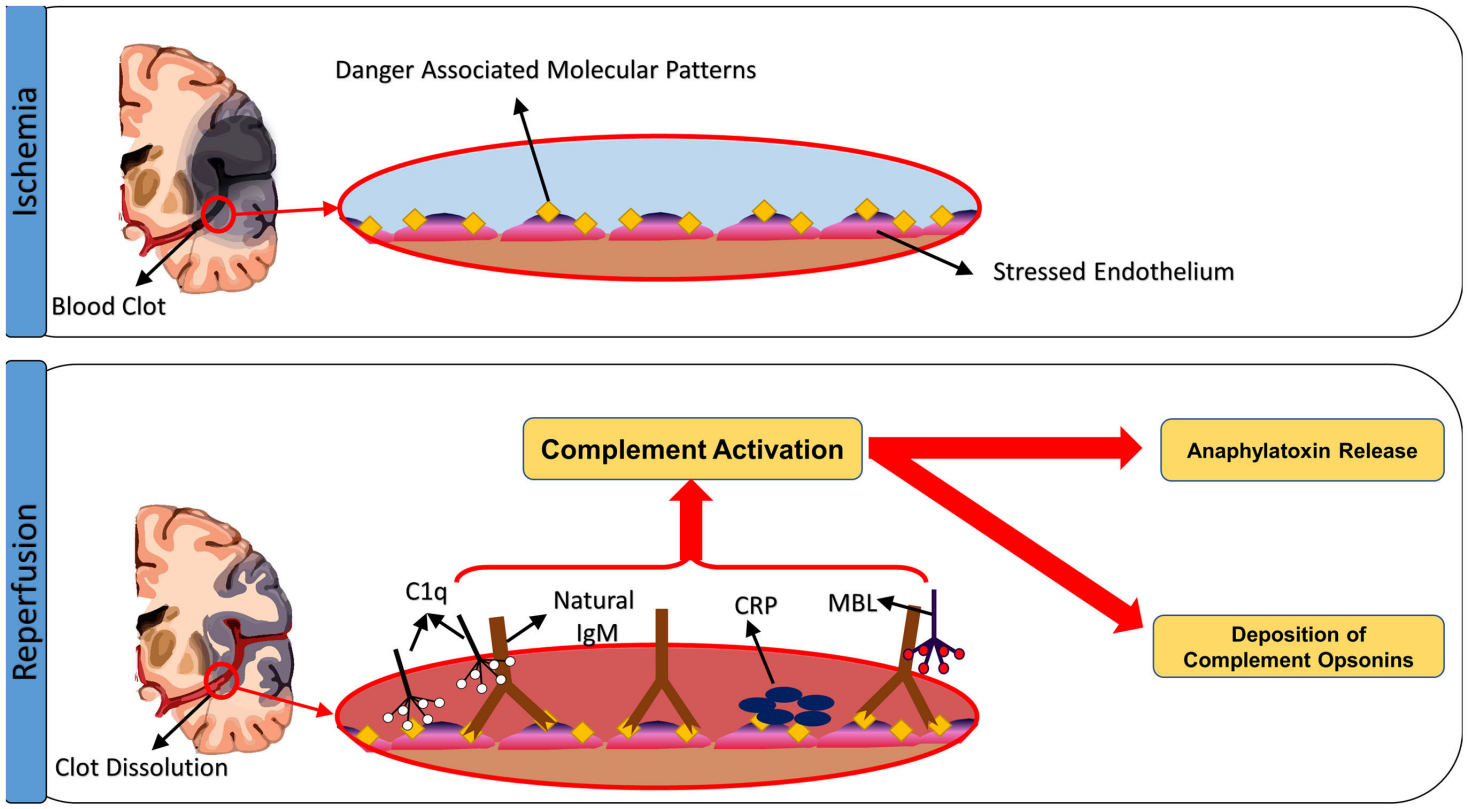
**Triggers of complement activation after cerebral ischemia-reperfusion injury**. Ischemic insult induces expression of neoepitopes or danger-associated molecular patterns (DAMPs) on the surface of stressed endothelial cells. The exposed DAMPs are recognized by circulating natural self-reactive antibodies, principally IgM, which triggers complement activation. Although IgM binds C1q, it appears to be the binding of MBL and activation of the lectin pathway that drives ischemia and reperfusion injury in the organs systems examined, including the brain. Complement can be also activated through direct binding of C1q to apoptotic cells, as well as through C-reactive protein-induced complement activation.

So what is the complement-activating event after stroke? Seminal studies by Williams et al. (97) and Zhang et al. (98, 99) demonstrated that following intestinal ischemia and reperfusion, complement is activated by natural self-reactive IgM antibodies that recognize post-ischemic neoepitopes. By screening IgM-producing hybridomas, an IgM monoclonal antibody was isolated that could restore pathogenic injury in otherwise protected antibody-deficient (Rag1^−/−^) mice. The intestinal target of this mAb was identified as non-muscle myosin (98), and antibodies to this post-ischemic neoepitope have additionally been shown to play a role in hind-limb (100) and myocardial IRI (101). Although the early studies attributed IgM-mediated activation of the classical pathway as an initiating event in IRI, more recent studies have demonstrated that IRI is driven by IgM-mediated activation of the lectin pathway, at least in the organ systems that have been investigated (99, 102, 103). The animal studies described above, and the human studies described below, indicate that the lectin pathway also initiates cerebral IRI, and recent studies have demonstrated that pathogenic natural IgM also plays a role in propagating injury in murine ischemic stroke (71) (Figure 3). As with intestinal IRI, Rag1-deficient mice are protected from cerebral IRI, and two IgM monoclonal antibodies were identified that reconstituted cerebral IRI in Rag1-deficient mice; these monoclonal antibodies recognized annexin IV and a subset of phospholipids, and thus identified danger-associated molecular determinants expressed after stroke in mice (71). Post-ischemic blockade of annexin IV or phospholipid neoepitopes thus represents a potential therapeutic strategy for inhibiting complement activation and reducing cerebral IRI. The same two post-ischemic neoepitopes are also expressed in the intestine (104) and heart [(57), Circulation, in press] after ischemia and reperfusion, indicating that similar pathophysiologically important epitopes recognized by IgM natural antibodies are expressed in different organs and tissues. Furthermore, it has been shown that human endothelial cells (HUVEC) express these two neoepitopes after exposure to hypoxia (71).

### Studies in human patients

There are no reported clinical trials on the use of complement inhibition in stroke, a direct consequence of the aforementioned conflicting pre-clinical data. Before an intervention can enter a clinical trial, stringent criteria recommended by the stroke therapy academic industry roundtable (STAIR) must be met. In brief, there should be consistent pre-clinical data in different animal species and in animal cohorts that represent the human stroke predisposed population. Such populations include both sexes, adult and elderly age groups, and patients with comorbidities such as hypertension and diabetes (105). Thus, studies on the role of complement in human stroke are correlational.

Studies on gene polymorphisms at complement gene loci, associated polymorphisms at the C5 and factor H gene loci with increased incidence of cerebrovascular accidents (106, 107). Among 16 single nucleotide polymorphisms (SNPs) at the C3 gene locus, two SNPs (rs2277984 and rs3745565) were associated with ischemic stroke (108). MBL genotype is also associated with stroke severity. In two cohorts of stroke patients, MBL-sufficient phenotypes were associated with larger infarct and less favorable outcome compared to MBL-deficient phenotypes (77, 109). Prospective studies on men with advanced atherosclerosis, higher levels of C4 and C5 are associated with the incidence of stroke (110). Similarly, a prospective study of 5850 healthy men showed that C4 and C3 levels correlated with other cardiovascular risk factors, and that very high C4 levels may predict the incidence of stroke (111). C4 was also shown to be a stroke predictor in men with known or suspected coronary artery disease (112). Compared to healthy volunteers, stroke patients were found to have higher plasma levels of C3a, C3, C4, C5, factor B, and the terminal complement complex (113–118). Serum levels of C3, C3c, and C4 were also associated with increased stroke severity in cardio-embolic stroke patients (118, 119). Despite these human studies that report alterations in serum complement levels in stroke patients and the association with outcome, the studies remain correlative and cannot demonstrate a definitive role for complement in the pathogenesis of stroke. Table 3 summarizes studies investigating complement system changes in patients with ischemic or hemorrhagic stroke.

**Table 3:** **Summary of studies performed in human patients investigating the role of complement in cerebral I/R**.

Study	Patient condition	Controls	Assay	Pathway	Relevant findings
(118)	Cardio-embolic and small vessel disease stroke (*n* = 79 each)	Matched control groups (*n* = 40 each)	ELISA for C3 and C3a levels	All pathways	(1) C3 and C3a were elevated acutely in both groups, and only C3 remained elevated at 3 months in both groups.(2) Phasic increase of C3 was associated with unfavorable outcome only in cardio-embolic patients
(120)	Coronary vessel disease, MI, and ischemic stroke (*n* ~55 each)	Matched controls	Immunofluorometric assay of MASP-1,2,3,4	Lectin pathway	(1) MASP-1 and MASP-2 levels were low in stroke patients. (2) MASP-3,4 levels were not different from controls
(107)	Carotid atherosclerosis (*n* = 1065)	None	Polymorphism in C5 gene	All pathways	Polymorphism of C5 gene was associated with increased plasma C5a levels and increased incidence of MACE in men
(121)	Fatal ischemic brain infarction (*n* = 10)	None	Immunohistochemistry of C9 deposition	Common terminal pathway	Infarct areas associated with increased C9 deposition and neutrophil infiltration
(113)	Acute stroke (*n* = 11)	Healthy volunteers (*n* = 9)	Serum levels of complement and inflammatory components	Common terminal pathway	(1) Terminal complement complex and CRP levels increased at 3–12 days after stroke compared to controls. (2) No significant change in soluble adhesion molecules and cytokines
(122)	Cerebrovascular disease (*n* = 292)	Healthy volunteers (*n* = 198)	Genotype of C4B alleles.	Classical pathway	There was significantly lower incidence of allo-type C4B1Q0 in patients with cerebrovascular disease compared to control
(119)	Cardio-embolic, thromboembolic, and lacunar stroke patients (*n* = 194)	NA	C3c and C4	Classical and alternative pathways	(1) Serum concentration of C3c and C4 was significantly reduced in cardio-embolic stroke compared to atherothrombotic and lacunar strokes. (2) Serum concentration reflects the stroke severity only in cardio-embolic stroke
(110)	Advanced atherosclerosis (*n* = 173)	None	C5a	All pathways	Complement activation with increased C5a levels is associated with higher risk of MACE.
(123)	Ischemic stroke (*n* = 80)	None	Platelet C4d levels	Classical pathway	Platelet C4d is associated with severe acute ischemic stroke.
(124)	Ischemic stroke (*n* = 26)	Severe carotid atherosclerosis (*n* = 26)	sC5b-9 and C4d levels	Classical and common terminal pathway	(1) sC5b-9 and C4d were higher in stroke patients. (2) Levels of sC5b-9 were associated with stroke severity and functional disability
(125)	Brains of stroke patients (*n* = 10)	Control brains (*n* = 10)	Postmortem complement levels.	Classical and common terminal pathway	Ischemic areas showed C1q, C4d, and C3c deposition as well as marked reduction in CD59 compared to normal tissue
(108)	Stroke patients (*n* = 844)	Healthy controls (*n* = 668)	Assay of 16 SNPs at C3 locus.	All pathways	Two SNPs at the C3 gene locus (rs2277984 and rs3745565) were associated with ischemic stroke
(116)	Stroke patients (*n* = 179)	Healthy controls (*n* = 156)	C3 and C4 plasma levels and complement hemolytic activity.	All pathways	Complement levels of C3 and C4 were elevated in stroke patients and associated with a worse neurological outcome.
(59, 60, 115)	Stroke patients (*n* = 16)	Healthy controls (*n* = 16)	C3a, C5a, and sC5b-9 levels in plasma	All pathways	(1) C3a was significantly elevated in stroke patients compared to controls till day 28. (2) C5a levels were elevated at 7–14 days. (3) sC5b-9 levels were significantly depressed in stroke patients till day 2.
(112)	Men with known or suspected coronary artery disease (*n* = 389)	None	C4 plasma levels	Classical pathway	Serum component C4 is predictor of stroke with known or suspected heart disease.
(111)	Initially healthy men (*n* = 5850)	None	C3 and C4 plasma levels.	Classical and common terminal pathway	(1) Serum C3 and C4 levels correlated with other cardiovascular risk factors. (2) Very high C4 levels may be associated with stroke incidence
(126)	First degree relatives to South Asian stroke patients (*n* = 143)	South Asian (*n* = 121) and white (*n* = 141) controls	C3, factor B, and CRP plasma levels	Alternative pathway	(1) No significant difference in C3, CRP, and factor B levels between South Asian first degree relatives and controls. (2) Only C3 and CRP levels were higher in South Asian groups compared to controls
(106)	Caucasians who developed a ischemic stroke (*n* = 235)	Controls (*n* = 235)	Complement factor H Y402H gene polymorphism	Alternative pathway	No association of Y402H polymorphism with risk of ischemic stroke
(117)	Cryptogenic stroke (*n* = 79) and large vessel disease stroke (*n* = 73)	Controls (*n* = 79)	Plasma C3 and C3a levels	All pathways	(1) C3 levels were elevated in stroke patients compared to controls. (2) Higher C3a levels were associated with stroke. (3) Plasma levels of C3 were associated with unfavorable outcome in large vessel disease patients at 3-month and 2-year interval.
(109)	Ischemic stroke (*n* = 353)	None	MBL plasma levels.	Lectin pathway	MBL deficiency is associated with smaller cerebral infarcts and favorable outcome in patients receiving conservative treatment.
(77)	Ischemic Stroke (*n* = 135) and hemorrhagic stroke (*n* = 26)	None	MBL genotype and plasma levels.	Lectin pathway	Unfavorable outcome was associated with MBL-sufficient genotype and MBL-circulating level
(127)	Patients with first ischemic stroke (*n* = 1451)	None	C3 and C4 plasma levels.	Terminal Pathway	C3 (not C4) levels of stroke patients on admission was independently associated with prognosis in male patients 3 months after the acute event
(128)	Patients with first ischemic stroke (*n* = 220)	Healthy controls (*n* = 100)	MBL plasma levels.	Lectin Pathway	MBL was an independent prognostic marker of functional outcome and death 90 days after stroke
(129)	Infants with hypoxic-ischemic encephalitis (*n* = 16)	Healthy controls (*n* = 7)	C9 and MAC levels in CSF.	Terminal pathway	(1) Infants with hypoxic-ischemic encephalitis had significantly higher MAC levels and significantly lower C9 levels in CSF compared to controls. (2) Postmortem staining of brain tissue of two infants revealed C9 deposition on morphologically apoptotic neurons.

## Future Perspectives on the Dual Role of Complements in Brain I/R

The predominant focus of studies on complement and stroke has been on the role of complement in perpetuating the inflammatory cascade and contributing to neuronal death. By comparison, very few studies have investigated the putative protective role that complement may play in ischemic rescue and ischemia-induced neurogenesis. The great majority of reports investigate acute-phase outcomes where complement blockade prevents activation of pro-inflammatory mechanisms. However, as indicated by a few studies, the same favorable outcomes may not occur in the subacute and chronic phases after stroke, when complement opsonins may be important for mediating anti-inflammatory events such as removal of debris, and when anaphylatoxins may contribute to homeostatic pro-survival and neuro-regenerative effects. It will be important to investigate how manipulation of the complement system impacts subacute and chronic outcomes, as well as to evaluate areas away from the ischemic core. Investigation of chronic outcomes may reveal a role for complement in maintaining neuronal survival after acute damage has been resolved, and possible mechanisms may include stimulation of neuronal growth factor production by astrocytes and microglia (35, 36), or reduction of glutamate-induced excitotoxicity (37–40). Stroke and brain injury are associated with a window of synaptic plasticity after injury that contributes to neurological recovery (88, 90). While various mechanisms have been implicated in this process, a role for complement has not been investigated. Based on the essential role of complement in synaptic pruning during CNS development, complement may also play a role in synaptic pruning and hippocampal plasticity after stroke, and may thus contribute to improved neurological outcome and facilitate rehabilitation.

Going forward, the design of experimental models to assess longer endpoints and multiple outcome measures after stroke may reveal a more diverse role for complement in ischemic injury and rescue. Future studies should also investigate the role of different complement components in early and delayed pathophysiological events. One area of investigation that has received little attention is the role of complement opsonins in early neuronal and synaptic loss after stroke, as well as the role of these opsonins in the resolution of inflammation and synaptic re-wiring that can occur later. Future studies should also address the interplay between complement opsonins and immune cells bearing receptors for these opsonins, especially infiltrating and resident macrophages/microglia; these opsonins may also play a role in driving macrophage polarization toward an anti-inflammatory and pro-regenerative phenotype. We also have an incomplete understanding of the role of the anaphylatoxins in stroke. They are implicated in recruiting and activating immune cells acutely, as well as stimulating neuronal genesis and migration in delayed phases after stroke. It is also not clear whether C3a and C5a have redundant, overlapping, or possibly divergent effects. Tools to address these questions are available in the form of genetically deficient mice and inhibitors that block the generation of specific complement activation products or that antagonize complement receptors, and it will be important for future studies to better evaluate long-term effects and outcomes after stroke.

## Clinical and Translational Perspective

As mentioned in Section “Studies in Human Patients,” polymorphism in complement genes is associated with ischemic stroke, and that acute stroke patients have higher serum levels of complement proteins compared to controls (Table 3). In addition, postmortem studies have identified both complement and IgM deposition in the human brain after stroke (125). This suggests that complement activation and deposition occurs in the human brain similarly to that in experimental models. Thus, given the correlation between complement activity and stroke outcome in human patients, the use of complement inhibition holds promise as a potential therapeutic intervention. Optimally, a complement inhibitor should effectively inhibit early pathogenic complement activation without depleting systemic complement activity in order to maintain the homeostatic and protective role of complement during recovery. A clear understanding of the role of different complement activation products and their spatial and temporal effects will be important to guide the development of an optimal anti-complement therapeutic strategy. As a final note, thrombolytic therapy using t-PA also results in complement activation (130), and it is possible that complement inhibition in the context of t-PA administration could provide a new adjuvant therapy to t-PA.

## Conflict of Interest Statement

The authors declare that the research was conducted in the absence of any commercial or financial relationships that could be construed as a potential conflict of interest.
